# Empowerment training to support service user involvement in mental health system strengthening in rural Ethiopia: a mixed-methods pilot study

**DOI:** 10.1186/s12913-022-08290-x

**Published:** 2022-07-08

**Authors:** Sisay Abayneh, Heidi Lempp, Sauharda Rai, Eshetu Girma, Medhanit Getachew, Atalay Alem, Brandon A. Kohrt, Charlotte Hanlon

**Affiliations:** 1 College of Health Sciences, School of Medicine, Department of Psychiatry, WHO Collaborating Centre in Mental Health Research and Capacity Building, Addis Ababa University, Addis Ababa, Ethiopia; 2 Madda Walabu University College of Education and Behavoural Studies, Robe, Ethiopia; 3 Centre for Rheumatic Diseases, School of Immunology and Microbial Sciences, Faculty of Life Sciences and Medicine, King’s College London, Weston Education Centre 10, Cutcombe Rd., London, SE5 9RJ UK; 4 Department of Psychiatry, George Washington University, Washington, DC USA; 5 Depatment of Preventive Medicine, School of Public Health, College of Health Sciences, Addis Ababa University, Addis Ababa, Ethiopia; 6 Centre for Innovative Drug Development and Therapeutic Trials for Africa (CDT-Africa), College of Health Sciences, Addis Ababa University, Addis Ababa, Ethiopia; 7 Centre for Global Mental Health, Institute of Psychiatry, Psychology and Neuroscience, King’s College London, 16 De Crespigny Park, London, SE5 8AF UK

**Keywords:** Training, PhotoVoice, Service user, Mental health, Sub-Saharan Africa

## Abstract

**Background:**

Increased service user involvement is recommended to improve weak mental health systems in low-and middle-income countries (LMICs). However, involvement is rarely implemented and interventions to support involvement are sparse. In this study we evaluated the acceptability, feasibility and perceived outcomes of an empowerment and training program for service users and health professionals to facilitate service user involvement in mental health system strengthening in rural Ethiopia.

**Methods:**

REducing Stigma among HealthcAreProvidErs (RESHAPE) is a training curriculum for service users, their caregivers and aspirational health workers, which uses PhotoVoice methodology, to prepare them in participation of mental health systems strengthening in LMICs. We delivered the RESHAPE training augmented with empowerment content developed in Ethiopia. The interactive face-to-face training was delivered to service users and caregivers (over 10 days), and health professionals (1 day) separately. The study was an uncontrolled, convergent mixed-methods design. The quantitative data consisted of process data, satisfaction questionnaire, and a retrospective pre-test survey. Qualitative data included exit and follow-up in-depth interviews with the service users. Descriptive statistics were performed for quantitative data, and qualitative data were thematically analysed. The findings were integrated through triangulation for convergent themes following analysis.

**Results:**

Twelve service users, 12 caregivers and 18 health professionals were enrolled, and completed the training. Participants valued the content and delivery process; the standard of the training program met their expectations and participation led to positive gains in understanding about mental illness, stigma, service-user involvement and human rights. The qualitative findings identified positive impacts, including increased self-confidence, sense of empowerment, social - and perceived therapeutic benefits.

**Conclusions:**

We found that the RESHAPE training with added content for Ethiopia, delivered using the PhotoVoice methodology, is feasible, acceptable and of value to develop and implement training programmes which can empower service users to be involved in mental health system strengthening in this setting. Further study to assess the impact on health systems strengthening is warranted.

**Supplementary Information:**

The online version contains supplementary material available at 10.1186/s12913-022-08290-x.

## Background

Empowering service users and caregivers (hereafter ‘service users’ unless otherwise specified) to be involved at all levels of the mental healthcare system (advocacy, policy, planning, service provision, monitoring, research and education) is at the centre of the international mental health policy agenda [1]. Involvement has been increasingly acknowledged as fundamental to the scale-up of quality mental health services in low-and middle-income countries (LMICs) [2–4]. However, actual involvement remains largely aspirational, with little guidance on how to achieve involvement in practice [2, 3, 5]. In many LMICs, service users are still largely excluded from involvement in mental health systems [2, 6]. Multiple factors hinder service user involvement, including the limited attention given to empowerment and mobilization of service users, and the lack of preparation of other stakeholders to work with service users [2, 6, 7].

There is a strong argument for interventions to build the capacity of service users and health professionals (including service managers) to facilitate active participation and to overcome stigma and exclusion [2, 8, 9]. Studies from high-income countries indicate that empowerment and training of key stakeholders is helpful and can lead to successful working partnerships [10–12]. However, the evidence base for effective models of capacity-building is weak, limited to descriptions of training initiatives, and there are no programmes to equip key stakeholders to work together within LMIC mental health systems [13–15].

In Ethiopia, there have been no training programs designed to equip service users and health professionals for collaborative working in mental health system strengthening. Indeed the key stakeholders themselves reported being ill-equipped and expressed a strong need for training on how to work together to effect change [16, 17]. As a part of an intervention to pilot a new model of service user involvement in mental health system strengthening in Ethiopia, the authors developed and delivered interactive training to service users and health professionals in the Sodo district of southern Ethiopia. In this paper, we describe the development of the pilot training program and evaluate the feasibility, acceptability, and perceived outcomes.

## Methods

### Study design

We used an uncontrolled, convergent mixed-method study design. This study formed part of a larger research project aiming to develop service-user involvement in mental health system strengthening in Ethiopia. This was initiated as part of the ‘Emerging mental health systems in low-and-middle-income countries’ (Emerald) project including six LMICs (Ethiopia, India, Nepal, Nigeria, South Africa and Uganda) [18]. In addition, this specific study was part of the OPAL (Optimizing Provider Attitudes and competence in Learning mental health systems) project [19], and was one component of an intervention to pilot a Theory of Change model for service user involvement in mental health system strengthening in rural Ethiopia, described elsewhere [17].

### Setting

The study was conducted in Sodo District of the Gurage Zone of the Southern Nations, Nationalities and Peoples’ Region, Ethiopia. The capital of the district, Buie, is located about 100 km south of Addis Ababa. At the time of the study, the district had 58 *kebeles* (sub-districts); four urban and 54 rural. The population of the district was about 170,000. Health care was provided by one primary hospital, eight primary health centres and 58 health posts.

### Participants

The participants of the study were service users, caregivers, and generic health professionals, purposively selected based on pre-set inclusion criteria (See Table 1). The service users had confirmed diagnoses of psychosis or epilepsy or alcohol use disorder or depression; and they all had accessed integrated mental healthcare in primary healthcare as part of a previous study implementing the World Health Organization mental health Gap Action Program (mhGAP) [20].

**Table 1:** Inclusion criteria for training participants

Inclusion criteria	Stakeholder groups		
	Service users	Caregivers	Health professionals
Health professional who directly provides healthcare to service-users			X
Have a psychiatrist-confirmed diagnosis of the WHO mental health Gap Action Program (mhGAP)‘priority’ mental health conditions: psychosis or epilepsy or alcohol use disorder or depression	X		
Immediate caregiver to the service-users and closely involved in care		X	
Adult(18^+^ years)	X	X	X
Considered by themselves, by healthcare providers, and by the family to have improved in capacity to participate/functioning and with stable mental health conditions	X		
Working or receiving/had received healthcare services from primary healthcare at the Sodo district for at least for six months	X	X	X
Participant has time available and is willing to be involved in the training, or share their own recovery stories	X	X	X

The clinician involved in the person’s care (a psychiatric nurse based in Buei Primary Hospital) recommended the service user participants based on his professional judgement. For participants who were not under the clinician’s immediate care, a project psychiatric nurse assessed their capacity to consent using a semi-structured approach used in the setting previously.

The health professionals were recruited from the Sodo district health office (the administrative office co-ordinating all non-hospital health care in the district) and three health facilities (Buie Hospital, Kela and Tiya primary centres) in the district. All participants were invited by formal letter to participate by the Sodo district health office and in collaboration with PRIME project field coordinators.

### Overview of the training program

#### Theoretical lens

The training program design and interactive delivery was informed by principles of participatory action research (PAR) [21] and experiential learning theories [22, 23], suitable for adult learning and for people with low literacy. Key characteristics of this approach were: (i) the attention paid to empowerment, building on strengths and resources inherent in participants, and being defined by mutual trust, co-learning, active participation, and respect to promote social action; (ii) the value placed on life experiences as a central and necessary part of the learning process, and (iii) acknowledgement that participants bring a wealth of experiential knowledge on the topics discussed and are well-positioned to speak about their experiences [21–23].

The REducing Stigma among HealthcAreProvidErs (RESHAPE) program is an initiative to engage service users and their caregivers to improve training of primary care workers in mental health services [15, 19]. RESHAPE was developed based on stigma reduction theories from social psychology, social neuroscience and medical anthropology using a ‘What Matters Most’ framework of motivation [15]. The core methodology is PhotoVoice, which is a participatory photographic narrative technique. PhotoVoiceis a PAR methodology, involving creation of a visual reality of a person’s lived experiences with a particular topic and their reflections. The PhotoVoice approach is rooted in participatory empowerment education/critical consciousness theory and other participatory approaches [24, 25]. These theoretical underpinnings promote power sharing, understanding of lived experiences and encourage the participants to construct and share their reality to take collective action for positive change. PAR PhotoVoiceh as been employed within the field of mental health to: (i) explore and understand service user experiences of treatment of mental illness and the recovery process, (ii) engage in psycho-education programmes, (iii) dispel issues such as shame, social exclusion and stigma associated with mental illnesses, and (iv) advocate for more inclusive, co-produced and participant-centric ways of uncovering and discovering the lived experiences of service users [24, 25]. Photovoice combines photography, interviews, and group discussions mainly guided by SHOWED questioning techniques to elicit discussion about participants photographs [24, 25]: What do you **S**ee here?;What is really **H**appening here?;How does this relate to **O**ur lives?;**W**hy does this condition **E**xist?;and What can we **D**o about it? For RESHAPE, PhotoVoice was used to help health workers understand the experience of mental health conditions through the eyes of persons with lived experience. RESHAPE was developed in Nepal, where service users and caregivers were trained in PhotoVoice and then presented the photography narratives to primary care workers participating in mhGAP training, local community groups and policy makers. Proof-of-concept testing has demonstrated the feasibility and acceptability of the approach in Nepal, as well as suggestive benefits for reducing stigma among primary care workers in mhGAP training [15]. The current study in Ethiopia is the first test of the RESHAPE approach outside of Nepal.

#### Development of the training program

The development of the training program was informed by identifying needs through in-depth qualitative interviews with service users, health professionals, planners and policy makers [16]. Potential approaches to address these needs were identified by mapping out existing materials, consulting with experienced researchers in LMICs, particularly informed by a training manual developed by a Nepal research team [15], and a review of the international literature on similar initiatives [10, 11].

Based on these inputs, co-authors (SA, CH, and HL) developed two training manuals for service users, and health professionals. The appropriateness, contextual relevance, basic delivery and assessment modalities for the translated manuals (Amharic) were reviewed by six professionals who were either university faculty staff members or researchers from diverse disciplines (psychiatry, health education, clinical psychology, public health, and mental health epidemiology). The core content of the training manual was similar for both target groups, but the service users training included additional content on basic knowledge about mental health and illness, treatment options and their rights to receive care. The training manuals are freely available [26].

The training program for service users had additional content on the development of recovery narratives using PAR PhotoVoice techniques based on RESHAPE [14, 15, 19]. The Ethiopian team had a close dialogue with the OPAL Nepal team who had first-hand practical experiences of providing training and applying PhotoVoice techniques.

#### Training delivery

The interactive training was delivered face-to-face separately for (i) health professionals, and (ii) service users. The training for health professionals was conducted for a full day, which was divided into seven sessions. The training for service-users consisted of two parts, including an initial three day training course that focused on core topics, and eight PAR photovoice sessions, each of 3–4 hours duration conducted two times per week. The photovoice training had two components. First, field activities whereby service-users were supplied with digital cameras, a notebook and pen to take photographs of people, places or objectives/mementos that depicted their own recovery journey, followed by preparation of service user narratives closely linked to the self-selected photos. Participants were first trained in the use of the digital cameras, including about the ethical aspects to consider when taking photographs of people. The participants then took photos for each session on three themes about the time before receipt of professional treatment, during treatment and after completing treatment or continuing to receive treatment.

Second, there were interactive classroom learning and reflection sessions. During the classroom sessions participants were asked to choose photographs that were most relevant to the theme and that they felt comfortable discussing in a group. The photographs were transferred onto laptops to display the photos for discussion with all participants. Accordingly, every service user presented and was involved in discussions about the photographs using the SHOWED techniques. A range of facilitated discussions were conducted on several topics, including identifying strengths and resources, combating stigma and myths about mental illness, advocacy, communication skills, understanding and managing crisis.

In line with principles of PAR and experiential learning theories, the learning goals, content, and activities for both target groups were designed and delivered consisting of interactive techniques, including didactic, small group discussions and presentations, case scenarios analysis, audio-visual material showing the recovery narratives from lived experiences in the local area, and own recovery narratives using PAR photovoice, and action plan development exercises.

The training for service user and health professionals groups was held in a private setting in Buie town in Amharic (the official language in the study district). However, there are diverse local languages spoken in the study site. Therefore, during the training, the psychosocial workers involved in the training assisted us by translating key concepts into the local languages for the specific training sites (Kistane Guragena). The first author (SA) with two research assistants led the training for health professionals. The training for service users was facilitated by SA and MG. A senior professor of psychiatry from Addis Ababa University (AA) taught the mental health related topics for service users. The trainers were assisted by three psychosocial service providers who lived in the study area. SA and MG had received a five-day intensive face-to-face training specific to facilitate the planned photovoice sessions by three professionals from Nepal. The OPAL Nepal team also provided ongoing supervision and support throughout the training.

#### Evaluation of the training program

We evaluated the training program using multiple sources of quantitative and qualitative data [27].

#### Quantitative assessment

The quantitative data collection consisted of self-reported or interviewer-administered (for those with no or low literacy) rating scales and training process data; which included demographic data, program feasibility and acceptability, and change in understandings of training content.

#### Demographic data

Socio-demographic data including gender, age, education, and experiences (work/caregiving or living with mental health condition) were gathered at the beginning of the first session.

#### Feasibilityand acceptability of the training program

To assess the feasibility of the training program, we collected process information that consisted of the number of participants who enrolled, attended and completed each training session. Acceptability of the program was operationalized as participants’ satisfaction with the training sessions, standard of the training and fulfilment of their expectations. This was assessed immediately after the completion of training using a questionnaire comprising both structured and unstructured questions [9]. Aspects of the training sessions were rated on a 4-point Likert scale from 1(excellent) to 4(poor), and questions about the standard of training and fulfilment of expectations were rated from1(strongly agree) to 4(disagree).

#### Evaluation of understanding

We employed a retrospective pre-post-test questionnaire to investigate whether the training produced changes in participants’ self-reported understanding related to the objectives of the training program. The concept of service user involvement is relatively new within the Ethiopian mental health system, and prior to this training participants had no experience of involvement and working collaboratively in this area [16]. We anticipated that in most cases participants lacked sufficient knowledge to provide pre-training ratings, as has been reported previously [28]. With the retrospective pre-post-test approach, participants estimate their pre-intervention levels of understanding training content after receiving the training and can therefore situate themselves in relation to what they have since learned. This avoids response-shift bias, which occurs when participants may not fully understand how to assess the target understandings before the training, therefore leading to inaccuracies in pre-test ratings [28, 29].

We employed a bespoke questionnaire developed for Emerald [9] in line with the training goals. The instrument consisted of 17 items for health professionals and 15 items for service-users, with 13 items common to both groups. Participants reported their response to the questions via a five-point scale of ‘not very well’ (1), ‘somewhat well’ (2), ‘don’t know’ (3), ‘moderately well’ (4), or ‘very well’ (5). The participants completed the questionnaire immediately at the end of the first day (health professionals) and after the three-day training (service users). For each item, participants provided two ratings (i) their current level understanding after the training, and (ii) retrospective estimate of their level before the training. Five interviewers wrote the responses for participants who had low literacy (*n* = 15) with their agreement.

### Qualitative assessments

#### Exit interviews

Face-to-face in-depth interviews were conducted in Amharic individually with the service users and caregivers (*n* = 24) immediately at the end of the last PhotoVoice session. The interviews explored why participants attended the training, the aspects of the program that they found most helpful and enjoyed, aspects that made them feel uncomfortable, how the program could be improved, and perceived benefits/impacts of the training. The interviews were conducted by five (2 female and 3 male) project field research assistants. The interviews were audio-recorded and lasted between 6 and 46 minutes (with an average of 13 minutes). In addition, qualitative responses to open-ended questions nested in the quantitative survey related to the perceived challenges or comforts about the training process were collected.

#### Follow-up in-depth interviews

Individual in-depth interviews were conducted in Amharic with subsets of service user (*n* = 4) and caregivers (*n* = 4) approximately 6 months after the end of the training sessions. In between the training and the interviews, each of the participants had gained practical experience of giving testimonies to health professionals on a five-day training programme (each participant had attended at least two training sessions). The topic guides were adapted from similar research used by the OPAL team. In addition to the exit interview questions, the topic guide aimed to explore participants’ impressions of the training, knowledge and skill they had acquired, and how they had applied the knowledge and the skills since the past training sessions. All interviews were conducted by a single experienced interviewer (female), who had not been engaged in the training programme. The interviews were conducted in Amharic and were audio-recorded. Interviews lasted between 16 and 58 minutes (with an average of 30 minutes).

#### Data analysis

We utilized a triangulated convergent mixed-methods approach [27, 30], whereby quantitative and qualitative data were collected concurrently or at follow-up, analyzed separately, and the results were triangulated to develop comprehensive understanding of the training program for the key stakeholders in relation to feasibility, acceptability and perceived benefits/impact.

#### Quantitative data

Descriptive statistics were used to analyze feasibility and acceptability assessments and improvements in understanding. Individual group scores/absolute numbers were calculated/ summarized, session attendance, completion of the program, and frequency of agreement (agree or strongly agree) for survey items. We did not perform inferential statistical testing on retrospective pre-test assessment, because of the small number of participants involved; rather we compared descriptive data.

#### Qualitative data

SA typed the responses to the open-ended questions and the data from process documentation to develop a dataset, and then open-coded and categorized the dataset into themes using inductive thematic analysis [31]. This analysis was augmented with the themes from interviews of the qualitative data. Qualitative data from exit and follow-up interviews were transcribed verbatim by independent transcribers and translated into English by research assistants from Addis Ababa University (*n* = 7) and SA (*N* = 21). Audio records of three exit interviews were not clearly audible and were excluded. The remaining transcripts (*n* = 19) of the exit interviews were included to develop codes, which ensured data saturation [32]. Transcripts were analyzed by SA using thematic inductive coding to uncover meaning in participants^’^ accounts of their involvement in the training process [31]. Details of narratives of service users’ recovery journey and challenges of living with mental illness will be reported separately.

#### Rigor

To enhance trustworthiness of the findings we employed different strategies, including data and methods triangulation, use of field observation notes, and prolonged engagement with participants [33]. We applied triangulation of multiple data sources that helped to include various points of data in the analysis. Through prolonged engagement in the field with participants, SA took observational field notes throughout the study period that helped to enrich data from the interview transcripts and compare personal experiences during the subsequently bracketing [34] and data analysis. Information for the photovoice narratives were participants’ own observations and interpretation included, which is considered as member checking [35].

## Results

The results of the training are presented in three sections, comprising participant characteristics, training program feasibility and acceptability, and perceived outcomes.

### Participant characteristics

Twelve service users, 12 caregivers and 18 health professionals attended and completed the training and provided response to the program assessments. Socio-demographic data for participants is presented in Table 2. To maintain anonymity, the participants were represented with identification numbers to describe their qualitative accounts in the results, for example service user (e.g. SU1, SU2), caregiver (e.g. CG1, CG2) or health professionals (HP1, HP2).

**Table 2:** Participants’ socio-demographic information

Characteristics	Service users	Caregivers	Health professionals
**Gender**			
Male	6	5	15
Female	6	7	3
**Age (years)**			
20–25	2	0	2
26–30	0	3	4
31–35	3	2	6
36–40	1	2	4
41+	6	5	2
**Educational attainment**			
Non-literate	4	7	0
Informal Education	2	2	0
Primary School	6	1	0
Secondary School	0	2	0
Diploma	0	0	5
First degree	0	0	13
**Years of work experience or caring or living with mental health condition**			
1-5 years	5	5	3
6-10 years	3	3	8
11-15 years	1	0	1
16^+^years	3	4	6

### Training program feasibility and acceptability

#### Feasibility

Feasibility of the training program was supported by high enrolment, training completion and response to the training assessment by all eligible service users (12/12), caregivers (12/12) and health professionals (18/20) (See Table 3). Moreover, all participants who enrolled attended all sessions, except one who missed one session because of a competing commitment.

**Table 3:** Training program feasibility and acceptability indicators

Indicators	Service uses	Caregivers	Health professionals
**Indicators of feasibility**			
Participants invited to participate in the training	12	12	20
Participants enrolled in training	12	12	18
Participants attended all the sessions	12	11	18
Participants completed the sessions	12	12	18
**Indicators of acceptability**			
Number of participants who reported that their expectations had been fulfilled:			
Strongly agree	10	11	12
Agree	2	1	6
**Number of participants who reported that the training of high standard:**			
Strongly agree	9	11	11
Agree	3	1	7

The analysis of the qualitative datasets (open-ended questions, interviews: exit and follow-up) strongly supported the feasibility of the training programme. The participants reported only a few challenges to attending the training program. These logistical issues included challenges with transportation, accommodation, overlap with their regular duties, and health challenges.

*I attended all the training attentively with interest. The time schedule was good and it did not waste our time. (SU20, Exit interview)*

*I attended all the 10 days, previously I used to feel health problems all the days, but there was no problem during the training. I saw changes [health] in this regard. (SU3, Exit interview)*

Only one participant (coming from a rural area) reported challenges related to transportation and weather conditions that affected attendance.

*Sometimes I was delayed getting transportation and when there was rain we did not get transport, but there was nothing else. (CG20, follow-up interview)*

Some participants coming from rural areas reported challenges related to accessing a power supply to charge the digital cameras. Notwithstanding the affirmative feedback about the training content and values, almost all health professionals indicated areas where the training programme could be improved in terms of time and supplementary reading materials.

*The training was very nice, but the time was very short. If the time was longer we could have discussed many points. The trainer covered a lot of key points in [a] very short time. (HP13, Response to open-ended questions)*

#### Acceptability

The quantitative analysis indicated that the majority of participants reported strong agreement (31/42) or agreement (11/42) that the training was of a high standard and that their expectations had been fulfilled (strongly agree = 33/42; agree = 9/42) (See Table 3). This positive feedback was captured in the qualitative data in participants’ accounts about the adequacy of the training content, recommendations about how the training could be improved, and the most useful aspects of the training.

Almost all participants mentioned that the training included many useful topics, and their expectations were largely met. They described the training program using words and phrases like ‘interesting’, ‘very nice’, ‘very relevant’, ‘very useful/helpful’, ‘very happy’, and one participant said “*This lesson cannot be gained even by paying for it,” (CG12-Follow-up interview).* Many of the participants recommended no change or additional topics to the training program, except three participants who wanted to additionally receive training about income generation (*SU11,* SU21,*CG4*).

*I am very happy about the training; the issues discussed were very important. The recommendations and solutions raised during the training should be changed into practice involving all stakeholders (HP6, Response to open-ended questions)*

*The training contained many useful topics. It helps to learn how to properly use medication, and how to teach other people. It helps to clearly learn from others’ stories, for example about how to avoid alcohol use and factors that worsen mental illness. (CG 22, Exit interview)*

Almost all participants recommended the need for expansion of the training programme to reach more people, including involving more people with lived experiences and engaging community stakeholders. The service users also expressed their readiness to share what they had gained from the training and their lived experiences.

*This training was provided for 12 service users and 12 caregivers. They should share the knowledge to many people. The training program should be supported by governmental and non-government organizations to reach many people. For example, if teachers participate in the training, the lesson can reach many people. (CG12. Follow-up interviews)*

The participants appreciated the different activities and interactive training techniques, including case scenarios analysis and PAR photovoice recovery narratives, which were considered to encourage collaborative learning, share experiences and made training understandable.

*The service users actively participated by capturing and describing photos and this made the training very clear to understand. This training included many things and was unusual. There were photographs, videos, and people with the mental health conditions shared their experiences throughout the training. I liked it very much. (CG 24, Exit interview)*

*The PhotoVoice was helpful to express idea easily. It nicely helped me to express my ideas. I am very happy talking about my experiences to others. (SU21, Exit interview)*

### Perceived outcomes of the training programme

#### Improved understanding/knowledge

The descriptive analysis of the retrospective pre-test responses showed that increased numbers of participants reported higher understanding levels after the training than before (see Additional file 1 and Table 4).

**Table 4:** Descriptive statistics of training program understanding after and before the training

	I understood:	Phases	N	Mean	St.d
1	Why service users and caregivers wanted to be involved in mental health systems strengthening	After	18	4.9	0.32
		Before		3.3	0.97
2	The value of service users and caregivers’ involvement in mental health system strengthening	After	18	4.9	0.32
		Before		3.3	1.09
3	How to involve service user and caregivers in the different aspects of mental health system strengthening	After	18	4.7	0.49
		Before		2.8	1.06
4	How to collaborate with service users and caregivers for mental health system strengthening	After	18	4.8	0.43
		Before		3.3	1.09
5	What kinds of contributions service users and their caregivers can make to improve mental care in my district (Sodo district)	After	42	4.6	0.59
		Before		3.0	1.08
6	About the international protections (and protections within Ethiopia) for the rights of people with mental health problems	After	42	4.3	0.67
		Before		2.9	1.30
7	The experiences of people with mental health conditions in Sodo district	After	42	4.3	0.75
		Before		2.7	1.27
8	The levels of service user and caregiver involvement in mental health system strengthening	After	42	4.3	0.75
		Before		2.8	1.17
9	Myths and facts about mental illness	After	42	4.6	0.59
		Before		3.4	1.23
10	Types of mental health related stigma and discriminations	After	42	4.6	0.55
		Before		3.3	1.05
11	Impacts of mental health related stigma and discrimination	After	42	4.6	0.54
		Before		3.4	1.08
12	Strategies to reduce mental health related stigma and discrimination	After	42	4.6	0.58
		Before		3.0	1.19
13	Types of mental health problems	After	42	4.7	0.47
		Before		3.6	1.08
14	Treatments that can help people with mental health problems	After	42	4.6	0.54
		Before		3.6	1.15
15	Definition of service user	After	42	4.7	0.48
		Before		3.2	1.09
16	Definition of caregiver	After	42	4.6	0.50
		Before		3.5	1.17
17	Definition of involvement in mental health system	After	42	4.6	0.55
		Before		3.3	1.13
18	How I can contribute to improve mental care in my district (Sodo district)	After	24	4.1	0.68
		Before		2.9	1.04
19	How I can contribute to the development of mental health policy and law development in Ethiopia	After	24	4.2	0.66
		Before		2.9	1.19

Consistent with the quantitative findings, the qualitative data supported those participants perceived this training improved their understanding. The participants reported that the PAR photovoice enhanced their active involvement and understanding of the training. The participants reported learning new skills that they could use or are using in various areas.

*The training gave us sufficient knowledge about mental illness, medication use and stigma and discrimination. I got good knowledge when I heard their advice (participants sharing lived experiences) and the way they talk. (SU16, Exit interview)*

*The training had a lot of benefit. When participants presented their experiences of living with mental illness, this becomes a good experience and advice for us [participants]. When they [service users] talk we [participants] were happy. Just when they speak about their past experiences and how well they are doing now it gives hope service user. (CG15, Follow-up interviews)*

Besides the improved understanding and skills, the qualitative data highlighted numerous perceived outcomes/benefits of the training programme, including feelings of empowerment, social benefits, and perceived therapeutic benefits.

#### Sense of empowerment

The participants mentioned the valuable contribution of their participation in the interactive PAR photovoice group activities and having the opportunity to give testimonies to health professionals. For many participants, the social space for sharing their recovery journey, having their views heard, valued and acknowledged by health professionals had led to improved self-confidence, a sense of valued contribution and increased self-worth.

*...they (health professionals) were very happy, clapped their hands for us, and they told us to help us [service users and caregivers] if we faced any problem even at night. (CG20, Follow-up interview)*

*I am very happy having a freedom to discuss with others and sharing my experience about my previous situation. I feel like I rebirth and feel like my age is just like a child. Previously I have suffered a lot … when that time passes and I become to this stage, this is rebirth for me. I am happy. (SU 17, Exit interview)*

#### Perceived social benefits

Almost all participants valued the social benefits of the training programme in terms of improved social acceptance. The participants mentioned the PAR photovoice process helped them to be valued and acknowledged for their lived experiences in the family and community.

*We were stigmatized and discriminated by community, no one used to visit us, including our neighbours, and we did not go to other peoples’ homes. After we started participation in this training many people started greeting us. My relatives who used to reject me are now accepting me. People started treating us as human beings. We have hope. God knows the future. (CG7, Exit interview)*

*In my family no one was looking for me because of my illness. They were wishing my death; no one used to give me even free rain water and considered me as useless. My husband is very nice person and he encouraged me and after my involvement in the training my family started visiting me. (SU11, Exit interview)*

*The training showed us many good things. I am communicating with my family very well now and they are also asking me to share my ideas with them. They are also accepting my ideas. (SU1, Exit interview)*

Although there was improved acceptance of service users in the community, the participants mentioned that more work needed to be done in the community and institutions through awareness creation and involving service-users.

*There are many people who stigmatise and discriminate against people with mental illness. They lack understanding about mental illness and people with mental illness. We should provide awareness to all people. (SU 11, Exit interview)*

At beginning of the training process, we observed that some participants felt uneasy about sharing their own recovery stories because of being upset due to past unpleasant experiences of discrimination. This problem was anticipated by the trainers before the training started and managed by probing participants to share their personal strengths rather than focus on their personal mental illness. The participants liked the opportunity to meet, spend time and interact with people with similar mental health conditions. The participants welcomed the opportunity to have the time and space to exchange their lived experiences and learn with or from each other. They reported that this created a sense of belonging, strengthened their relationships and stimulated action to establish long-term relationships through getting organized into a grass roots service user association. The participants reported that being organized into an association would support their collective efforts to tackle problems facing people with mental health conditions.

*The training process brings people to deeply connect with each other. Our (service users) coming and discussing together enabled us to establish a service users association, and hope that it [association] will be the best association in the Sodo district and the entire country. (CG12, Exit interview)*

#### Perceived therapeutic benefits

Many of the participants reported making changes in their lifestyles and behaviour with perceived therapeutic value because of the knowledge and skills gained from the training. Some reported making changes to unhealthy aspects of their lives, including reducing/stopping alcohol drinking, improved medication use, attending health facilities, improvements in the way they experienced their family relationships and the wider community.

*The man (service user) used to be in bed and feeling pain all day; and I was not able to attend meeting even at my neighbourhood. I am very happy for attending this training. The man is effectively taking his medication. The man was a difficult person, but after the training [he] has showed improvement. He started meeting people and participation in social activities. He is started looking for and feeding cattle. The training gives hope. (CG2, Exit interview)*

*This training was more than taking medication, helpful to change the mind and giving hope about the future. Thanks to God we are here to get involved here. We used to chain her all the days just like a sheep and goat. I remember many people visited and took her photo while she was chained. (CG7, Exit interview)*

Many of the service users mentioned involvement in peer support, advocating for human-rights and supportive relationships with health service providers that could help to improve health service quality.

*When both of us (service user and caregiver) go to health facility, we don’t feel shameful and [can] freely talk about her (service user) mental health condition without fear. I ask for timely service without waiting for long. They (health professionals) are also serving us with good manner and respect. (CG10, Exit interview)*

## Discussion

In this paper we have described the development, delivery and evaluation of the feasibility, acceptability, and perceived impacts of a participatory training programme for mental health service users and health professionals. This was one component of an intervention to support service user involvement in mental health system strengthening in Ethiopia [17]. There was high participant enrolment, completion, response to assessments, and few challenges or discomforts during the training process. The training program content, delivery process, standard, and relevance were well received. The participants recommended continuing and expanding the training program to reach many people**.** The training had positive impacts, including improved understanding of issues related to service user involvement, mental illness, increased confidence, social impacts and perceived therapeutic benefits. Our program showed that training service users and health professionals for greater involvement in mental health system strengthening is feasible, acceptable, and can have several benefits at small scale in rural Ethiopia.

The preliminary findings of feasibility and acceptability of the training programme indicate that this is a promising approach to equip key stakeholders to support greater service user involvement [17, 36]. This step was identified as a necessary precondition to service user involvement in our Theory of Change model for mental health system strengthening in Ethiopia [17]. This study also contributes to the small evidence base on training programs designed to empower mental health service users in LMICs [5, 8, 9, 13].

Our findings indicated that participants valued the wide range of interactive training strategies utilized during the training (e.g., group exercises and reflections, photovoice recovery narratives). The findings reinforce studies from high-income countries that reported similar active involvement of service-users [11, 12]. These activities are key strengths of PAR and experiential learning approaches that we applied, which integrate participation, promote supportive relationships, and recognise knowledge and lived experiences as vital to the knowledge generation process [22, 23, 37]. Many publications report that the PAR photovoice method values the expertise of participants and their direct involvement in all aspects of research is positive, including the visual data analysis about the meaning and importance of self-selected of photos and their narratives [24, 25, 38]. PhotoVoice is inclusive and particularly suitable for those with low literacy, because this method does not presuppose any working knowledge of traditional research methods; and participants mainly use photography as the medium through which they communicate their lived experiences [24, 25]. Combined use of visual data (photos), together with individual and group reflective sessions (voice), can facilitate an authentic, active participation, co-creating/co-production of knowledge, and provides a powerful means for marginalized groups to communicate their experiences [25, 38].

Our findings show that the training program positively improved participants’ understandings related to service user involvement, and mental health recovery journeys. This is in line with evidence that show that PhotoVoice approaches support participants to take an active role and improve understanding by conveying the why, how, and what of living with mental illness, the complexities of their recovery process, and their experiences of dealing with mental health challenges in the private and public domains [25, 39]. In addition, through the active involvement of participants in the PhotoVoice process by learning new skills (e.g. photography, collaborative group working) can promote increased self-confidence, self-worth, and sense of contribution [25, 39].

Moreover, we identified that service users reported the adoption of more healthy lifestyles in a number of ways, for example, reduced alcohol consumption, effective use of medication, and improved social interaction and healthcare attendance. These outcomes are in line with evidence that greater participation in PhotoVoice can have therapeutic benefits for participants to improve self-understanding, a sense of empowerment, self-esteem, and assistance to overcome stigma [25, 40]. Similarly, case studies from diverse countries reported that programmes that engage service users to share their recovery narratives can reduce stigma, facilitate healing, and promote engagement in mental healthcare, while also assuring that service users have autonomy in the content and form of the narratives [41].

Our results show that the participants reported improved social acceptance in their community. This is consistent with the evidence that active participation and reflection during photovoice can achieve social acceptance through addressing multi-level stereotypes, stigma and feeling of shame and isolation that is often associated with mental illness [25, 38]. This ‘social contact’ aspect of PhotoVoice in challenging stigma is likely to be crucial in overcoming the main barrier that service users face to meaningful civic participation and steers away from the past systemic disadvantages in most facets of their daily lives in LMICs [42, 43].

The training process had social benefits, including opportunities to meet and engage with people with similar experiences, share experiences and learn from each other, and building social networks. Others studies also reported that such sharing of stories can instil a sense of connectedness, and that participants benefit from group support due to the shared experiences [25, 39, 44]. In our study, service users who participated in the training decided to establish a service user association in Sodo district. The association comprised of 24 founding members (12 service users and 12 caregivers) to support each other to expand their networks in an organized way for involvement in mental health systems strengthening and to advocate for their human rights.

We also identified areas of the training programme that need further attention. Health professionals suggested future training might be improved by extending the one-day program to facilitate in-depth coverage of the training content. Our training process observations show that some service users were highly emotional when narrating their recovery journey. This implies the importance of taking necessary precautions and preparation of participants for strategic disclosure of recovery narrative during the PAR photovoice process. Other studies also recommended the need for preparatory training (e.g. “Coming Out Proud”) to support participants to affirm their own readiness to disclose their mental illness and combat self-stigma and reframe the meaning of mental illness [45].

### Strengths and limitations

The program development and delivery were informed by participatory action research and an experiential learning approach that promoted active involvement of participants. We employed a prospective convergent mixed methods design, which utilized multiple data sources and allowed us to triangulate findings.

Nonetheless, there were several limitations of this pilot study that warrant consideration. (i) The study sample was small, (ii) there was no control group and (iii) participants were selected purposively, with the potential for selection bias that may limit generalizability of the findings. There was also concern that an (iv) interviewer administered assessment could lead to social desirability bias. The quantitative measures used in this study, although based on other studies of a similar nature, have undergone limited psychometric testing or were unstandardized measures with unknown psychometric properties. We employed a PAR real-time engagement approach and several triangulation techniques to obtain in-depth assessment of the perceived understanding changes, however, use of validated measures in future evaluation of this training will be useful in benchmarking improvements against other training approaches. Moreover, this study did not evaluate the impact on health workers attitudes and behaviors, which are the end targets of the RESHAPE intervention.

## Conclusions

Our study has provided preliminary evidence of the acceptability, feasibility and perceived benefits of a training programme to empower and equip service users and health workers to collaborate to strengthen mental health systems in rural Ethiopia. Future work needs to focus on evaluation of the programme at a larger scale and to further investigate the ongoing support required to achieve sustainable service user involvement.

## Supplementary Information

**Additional file 1.**

## Data Availability

The datasets generated during and/or analysed during the current study is available from the corresponding author on reasonable request.

## Abbreviations

CAB: Community Advisory Board
Emerald: Emerging mental health systems in low- and middle-income countries
LMICs: Low-and-middle-income countries
mhGAP: Mental Health Gap Action Programme
RESHAPE: REducing Stigma among HealthcAreProvidErs
OPAL: Optimizing Provider Attitudes and competence in Learning mental health systems
PAR: Participatory Action Research
PRIME: PRogramme for Improving MentalhealthcarE
RAG: Research Advisory Groups
RPG: Research ParticipantGroups
ToC: Theory of Change
