# Supplementary material for: Empowerment training to support service user involvement in mental health system strengthening in rural Ethiopia: a mixed-methods pilot study
Source: BMC Health Serv Res. 2022 Jul 8;22:880. doi: 10.1186/s12913-022-08290-x (PMC9264546; doi:10.1186/s12913-022-08290-x)
Supplement: Supplementary file 1 — Additional file 1. [file 12913_2022_8290_MOESM1_ESM.docx]

***Appendix 1. Frequencies of participants’ response to the topic after the training and before the training***

Not very well (N) 2. Somewhat well (S) 3. Don’t know (D) 4. Moderately well (M) 5. Very well (V)

|  | I understood: | Phases | Service users | | | | | Caregivers | | | | | Health professionals | | | | |
| --- | --- | --- | --- | --- | --- | --- | --- | --- | --- | --- | --- | --- | --- | --- | --- | --- | --- |
|  |  |  | N | S | D | M | V | N | S | D | M | V | N | S | D | M | V |
| 1 | Why service users and caregivers wanted to be involved in mental health systems strengthening | After |  |  |  |  |  |  |  |  |  |  | 0 | 0 | 0 | 2 | 16 |
|  |  | Before | - | - | - | - | - | - | - | - | - | - | 5 | 3 | 9 | 1 | 0 |
| 2 | The value of service users and caregivers’ involvement in mental health system strengthening | After |  |  |  |  |  |  |  |  |  |  | 0 | 0 | 0 | 2 | 16 |
|  |  | Before | - | - | - | - | - | - | - | - | - | - | 6 | 2 | 8 | 2 | 0 |
| 3 | How to involve service user and caregivers in the different aspects of mental health system strengthening | After |  |  |  |  |  |  |  |  |  |  | 0 | 0 | 0 | 2 | 16 |
|  |  | Before | - | - | - | - | - | - | - | - | - | - | 1 | 9 | 1 | 7 | 0 |
| 4 | How to collaborate with service users and caregivers for mental health system strengthening | After |  |  |  |  |  |  |  |  |  |  | 0 | 0 | 0 | 6 | 12 |
|  |  | Before | - | - | - | - | - | - | - | - | - | - | 2 | 2 | 2 | 12 | 0 |
| 5 | What kinds of contributions service users and their caregivers can make to improve mental care in my district (Sodo district) | After | 0 | 0 | 1 | 5 | 6 | 0 | 0 | 0 | 6 | 6 | 0 | 0 | 1 | 4 | 13 |
|  |  | Before | 3 | 2 | 5 | 2 | 0 | 1 | 1 | 6 | 4 | 0 | 1 | 6 | 1 | 9 | 1 |
| 6 | About the international protections (and protections within Ethiopia) for the rights of people with mental health problems | After | 0 | 0 | 1 | 8 | 3 | 0 | 0 | 0 | 8 | 4 | 0 | 1 | 1 | 7 | 9 |
|  |  | Before | 3 | 4 | 3 | 1 | 1 | 2 | 0 | 4 | 6 | 0 | 4 | 3 | 1 | 8 | 2 |
| 7 | The experiences of people with mental health conditions in Sodo district | After | 0 | 0 | 2 | 6 | 4 | 0 | 0 | 1 | 8 | 3 | 0 | 1 | 1 | 4 | 12 |
|  |  | Before | 4 | 2 | 3 | 3 | 0 | 2 | 4 | 1 | 5 | 0 | 3 | 5 | 2 | 6 | 2 |
| 8 | The levels of service user and caregiver involvement in mental health system strengthening | After | 0 | 0 | 3 | 5 | 4 | 0 | 0 | 3 | 4 | 5 | 0 | 0 | 1 | 5 | 12 |
|  |  | Before | 2 | 5 | 4 | 1 | 0 | 2 | 3 | 3 | 3 | 1 | 2 | 6 | 1 | 8 | 1 |
| 9 | Myths and facts about mental illness | After | 0 | 0 | 2 | 6 | 4 | 0 | 0 | 0 | 6 | 6 | 0 | 0 | 0 | 2 | 16 |
|  |  | Before | 2 | 4 | 2 | 4 | 0 | 1 | 1 | 3 | 7 | 0 | 0 | 4 | 0 | 7 | 7 |
| 10 | Types of mental health related stigma and discriminations | After | 0 | 0 | 0 | 8 | 4 | 0 | 0 | 1 | 4 | 7 | 0 | 0 | 0 | 5 | 13 |
|  |  | Before | 1 | 2 | 4 | 5 | 0 | 1 | 1 | 3 | 7 | 0 | 0 | 6 | 0 | 9 | 3 |
| 11 | Impacts of mental health related stigma and discrimination | After | 0 | 0 | 1 | 5 | 6 | 0 | 0 | 0 | 5 | 7 | 0 | 0 | 0 | 4 | 14 |
|  |  | Before | 1 | 5 | 2 | 4 | 0 | 0 | 2 | 3 | 6 | 1 | 0 | 4 | 2 | 8 | 4 |
| 12 | Strategies to reduce mental health related stigma and discrimination | After | 0 | 0 | 2 | 5 | 5 | 0 | 0 | 0 | 4 | 8 | 0 | 0 | 0 | 3 | 15 |
|  |  | Before | 4 | 3 | 2 | 3 | 0 | 0 | 4 | 1 | 7 | 0 | 1 | 5 | 2 | 8 | 2 |
| 13 | Types of mental health problems | After | 0 | 0 | 0 | 6 | 6 | 0 | 0 | 0 | 3 | 9 | 0 | 0 | 0 | 4 | 14 |
|  |  | Before | 1 | 1 | 5 | 5 | 0 | 1 | 0 | 1 | 9 | 1 | 1 | 2 | 1 | 8 | 6 |
| 14 | Treatments that can help people with mental health problems | After | 0 | 0 | 1 | 5 | 6 | 0 | 0 | 0 | 6 | 6 | 0 | 0 | 0 | 4 | 14 |
|  |  | Before | 1 | 3 | 3 | 5 | 0 | 1 | 1 | 1 | 7 | 2 | 1 | 2 | 1 | 9 | 5 |
| 15 | Definition of service user | After | 0 | 0 | 0 | 6 | 6 | 0 | 0 | 0 | 5 | 7 | 0 | 0 | 0 | 3 | 15 |
|  |  | Before | 1 | 6 | 2 | 3 | 0 | 0 | 3 | 3 | 5 | 1 | 0 | 5 | 1 | 9 | 3 |
| 16 | Definition of caregiver | After | 0 | 0 | 0 | 8 | 4 | 0 | 0 | 0 | 7 | 5 | 0 | 0 | 0 | 2 | 16 |
|  |  | Before | 1 | 4 | 1 | 5 | 1 | 1 | 3 | 1 | 6 | 1 | 0 | 3 | 0 | 10 | 5 |
| 17 | Definition of involvement in mental health system | After | 0 | 0 | 1 | 7 | 4 | 0 | 0 | 0 | 7 | 5 | 0 | 0 | 0 | 2 | 16 |
|  |  | Before | 1 | 6 | 1 | 3 | 1 | 1 | 2 | 3 | 6 | 0 | 0 | 4 | 1 | 10 | 3 |
| 18 | How I can contribute to improve mental care in my district (Sodo district) | After | 0 | 0 | 3 | 6 | 3 | 0 | 0 | 1 | 7 | 4 | - | - | - | - | - |
|  |  | Before | 2 | 3 | 5 | 2 | 0 | 1 | 2 | 3 | 6 | 0 | - | - | - | - | - |
| 19 | How I can contribute to the development of mental health policy and law development in Ethiopia | After | 0 | 0 | 2 | 8 | 2 | 0 | 0 | 1 | 5 | 6 | - | - | - | - | - |
|  |  | Before | 3 | 2 | 4 | 3 | 0 | 1 | 3 | 2 | 5 | 1 | - | - | - | - | - |
